# Texas State Medical Association; Announcement of Officers of Sections for 1896-7*The Secretary, Dr. H. A. West, has kindly furnished the Journal with the list of appointments of Section officers, just completed.—Ed.]

**Published:** 1896-07

**Authors:** H. A. West

**Affiliations:** Sec’y


### Society Notes.

For the Texas Medical Journal.

### Texas State Medical Association.

### ANNOUNCEMENT OF OFFICERS OF SECTIONS FOR 1896–7.*

The committee consisting of the President and the Vice-Presidents of the State Medical Association, has made the following appointments:

#### SECTION ON GENERAL MEDICINE.

Chairman, Dr. L. Ashton, Dallas; Secretary, Dr. H. L. Tate, Lindale.

*The Secretary, Dr. H. A. West, has kindly furnished the Journal with the list of appointments of Section officers, just completed.—Ed.]

SECTION ON OBSTETRICS AND DISEASES OF WOMEN.

Chairman, Dr. A. M. Douglas, Osceola; Secretary, Dr. R. L. Miller, Denton.

SECTION ON SURGERY.

Chairman, Dr. Bacon Saunders, Fort Worth; Secretary, Dr. A. C. Scott, Temple.

SECTION ON MEDICAL JURISPRUDENCE.

Chairman, Dr. T. J. Bennett, Austin; Secretary, Dr. F. S. White, Terrell.

SECTION ON STATE MEDICINE AND PUBLIC HYGIENE.

Chairman, Dr. T. M. Cline, Galveston; Secretary, J. A. Mullen, Houston.

SECTION ON GYNECOLOGY.

Chairman, Dr. R. R. Walker, Paris; Secretary, Dr. J. B. Kirkpatrick, Comanche.

SECTION ON OPHTHALMOLOGY AND OTOLOGY.

Chairman, Dr. Van H. Hulen, Galveston; Secretary, Dr. R. F. Miller, Sherman.

SECTION ON DERMATOLOGY.

Chairman, Dr. Geo. H. Lee, Galveston; Secretary, Dr. W. M. Yates, Grandview.

SECTION ON MICROSCOPY AND PATHOLOGY.

Chairman, Dr. W. R. Blailock, McGregor; Secretary, Dr. W. F. Starley, jr., Galveston.

COMMITTEE ON STATE BOARD OF HEALTH.

Chairman, Dr. E. A. Woldert, Tyler; Secretary, Dr. F. A. Young, Navasota.

COMMITTEE ON NECROLOGY.

Chairman, Dr. F. E. Daniel. Austin; Secretary, Dr. B. F. Brittain, Arlington.

COMMITTEE OF ARRANGEMENTS.

Chairman, Dr. R. R. Walker, Paris.

The President has appointed the following:

COMMITTEE ON MEDICAL SOCIETIES.

W. R. Blailock, McGregor, Chairman; E. A. Woldert, Tyler; L. L. Shropshire, San Antonio; Joe D. Becton, McKinney; S. E. Hudson, Austin.

As regards delegates to the Pan-American Medical Congress, at the City of Mexico, in November, those who wish to attend will be appointed on making application to the President or Secretary.

H. A. West, Sec'y.

### Mississippi Valley Medical Association.

H. O. Walker, M. D., President, Detroit, Mich.; Merrill B. Ricketts, M. D., First Vice President, Cincinnati, O.; William F. Barclay, M. D., Second Vice President, Pittsburg, Penn.; H. W. Loeb, M. D., Secretary, 3559 Olive St., St. Louis, Mo.; Harold N. Moyer, M. D., Treasurer, Chicago, Ills.; C. A. Wheaton, M. D., Chairman Committee of Arrangements, St. Paul, Minn.

Executive Committee.—Wm. N. Wishard, M. D., Indianapolis; X. C. Scott, M. D., Cleveland; Geo. J. Cook, M. D., Indianapolis; J. M. Mathews, M. D., Louisville; I. N. Love, M. D., St. Louis; A. M. Owen, M. D., Evansville; Wm. T. Belfield, M. D., Chicago; C. S. Cole, M. D., New York; C. A. L. Reed, M. D., Cincinnati; R. Stansbury Sutton, M. D., Pittsburg.

The Twenty-second annual meeting of the Mississippi Valley Medical Association will be held at St. Paul, Minn., October 20, 21, 22 and 23, 1896. You are cordially invited to attend.

The meeting promises to be the largest in the history of the Association. Many valuable papers will be presented, and we hope that you may find time to prepare a paper. If you conclude to do so, please send the title to H. W. Loeb, M. D., 3559 Olive Street, St. Louis, Mo., or myself, at an early date.

Yours truly,

H. O. Walker, President.

H. W. Loeb, Secretary.

P. S.—We hope to get one fare for the round trip. Write to X. C. Scott, M. D., Chairman of Committe on Transportation, 457 Prospect Street, Cleveland, Ohio.

